# Surfactant Protein A and B Gene Polymorphisms and Risk of Respiratory Distress Syndrome in Late-Preterm Neonates

**DOI:** 10.1371/journal.pone.0166516

**Published:** 2016-11-11

**Authors:** Maria-Eleni I. Tsitoura, Eleana F. Stavrou, Ioannis A. Maraziotis, Kosmas Sarafidis, Aglaia Athanassiadou, Gabriel Dimitriou

**Affiliations:** 1 Neonatal Intensive Care Unit, Department of Pediatrics, Faculty of Medicine, University of Patras, Rio, Patras, Greece; 2 Department of General Biology, Faculty of Medicine, University of Patras, Rio, Patras, Greece; 3 First Department of Neonatology, Aristotle University of Thessaloniki, Ippokration General Hospital, Thessaloniki, Greece; University Children's Hospital Bern, SWITZERLAND

## Abstract

**Background and Objectives:**

Newborns delivered late-preterm (between 34^0/7^ and 36^6/7^ weeks of gestation) are at increased risk of respiratory distress syndrome (RDS). Polymorphisms within the surfactant protein (SP) A and B gene have been shown to predispose to RDS in preterm neonates. The aim of this study was to investigate whether specific SP-A and/or SP-B genetic variants are also associated with RDS in infants born late-preterm.

**Methods:**

This prospective cross-sectional study included 56 late-preterm infants with and 60 without RDS. Specific SP-A1/SP-A2 haplotypes and SP-B Ile131Thr polymorphic alleles were determined in blood specimens using polymerase-chain-reaction and DNA sequencing.

**Results:**

The SP-A1 6A^4^ and the SP-A2 1A^5^ haplotypes were significantly overrepresented in newborns with RDS compared to controls (OR 2.86, 95%CI 1.20–6.83 and OR 4.68, 95%CI 1.28–17.1, respectively). The distribution of the SP-B Ile131Thr genotypes was similar between the two late-preterm groups. Overall, the SP-A1 6A^4^ or/and SP-A2 1A^5^ haplotype was present in 20 newborns with RDS (35.7%), resulting in a 4.2-fold (1.60–11.0) higher probability of RDS in carriers. Multivariable regression analysis revealed that the effect of SP-A1 6A^4^ and SP-A2 1A^5^ haplotypes was preserved when adjusting for known risk or protective factors, such as male gender, smaller gestational age, smaller weight, complications of pregnancy, and administration of antenatal corticosteroids.

**Conclusions:**

Specific SP-A genetic variants may influence the susceptibility to RDS in late-preterm infants, independently of the effect of other perinatal factors.

## Introduction

Late-preterm newborns, defined as those delivered between 34^0/7^ and 36^6/7^ weeks of gestation, are at increased risk of adverse respiratory outcomes compared to their full-term counterparts [1]. Respiratory distress syndrome (RDS) remains the leading cause of acute respiratory morbidity in this population [2]; the disease has been attributed to a transient, qualitative and/or quantitative deficiency of pulmonary surfactant superimposed upon a relatively immature lung [3, 4].

Pulmonary surfactant is a lipid-protein complex which lines the alveolar surface and prevents alveolar collapse at expiration. Four surfactant proteins (SP), SP-A, SP-B, SP-C and SP-D, have been recognized [5]. The SP-A is a hydrophilic protein which participates in several processes, mainly related to innate immunity and surfactant function [6, 7]. The human SP-A gene locus is located on chromosome 10 10q22-q23), and consists of two functional and quasi identical genes, the SFTPA1 (SP-A1) and SFTPA2 (SP-A2), approximately 40 kb pair apart [8]. Combinations of single nucleotide polymorphisms (SNPs) within the coding region of these genes form intragenic haplotypes which determine more than 30 genetic variants [9]; however, four SP-A1 haplotypes (coded 6A, 6A^2^, 6A^3^, 6A^4^) and six SP-A2 haplotypes (coded 1A, 1A^0^, 1A^1^, 1A^2^, 1A^3^, 1A^5^) are the most prevalent [10]. The SP-B is a hydrophobic protein expressed in the alveolar type II epithelial cells; it is encoded by a single gene (SFTPB) located on chromosome 2 and is primarily involved in the structure and function of the surfactant [11]. Hereditary SP-B deficiency is lethal, as it leads to RDS that is refractory to surfactant replacement therapy [12].

A considerable body of evidence suggests an association between SP-A and SP-B genetic polymorphisms and development of RDS in preterm neonates [13–19]. Specific SP-A haplotypes have been shown to predispose to RDS while others have a protective effect, albeit with significant ethnical and racial differences [13–16]; an association between RDS and SP-B polymorphisms, such as the SP-B Ile131Thr polymorphism and the Δi4 length variation, has also been suggested [14,17–19]. Although the molecular mechanisms underlying these associations remain unknown, specific SNPs that result in amino acid substitution may alter the SP-A/SP-B structure and function, and contribute to the development of RDS under certain perinatal conditions [20]. To the best of our knowledge, there is a paucity of data in the literature regarding similar genetic contributors of RDS in the more ‘mature’ late-preterm newborns.

The aim of the present study was to investigate whether specific SP-A1/SP-A2 haplotypes and/or SP-B Ile131Thr polymorphic alleles are associated with RDS in infants born late-preterm, after taking into account the effect of other perinatal risk or protective factors [21].

## Methods

### Study population and protocol

This cross-sectional study included newborns delivered from 34^0/7^ to 36^6/7^ weeks of gestation and cared for at the University Hospital of Patras, Greece, between January 2011 and December 2012. The gestational age of the neonates was determined based on a combination of the early second-trimester ultrasound and the date of the last menstrual period, and was confirmed by the Expanded New Ballard Score. When the date of the last menstrual period was in doubt or when there was a discrepancy between the calculated dates, the determination of gestational age was solely based on the early second-trimester ultrasound.

The study was strictly adhered to the protocols applied in our institution for late-preterm neonates with respiratory distress; the assignment of any related medical intervention was at the discretion of the attending neonatologists. Late-preterm infants with moderate respiratory distress (respiratory rate >60 per minute, retractions, expiratory grunt), as well as those with mild respiratory distress but unable to maintain arterial oxygen saturation levels above 94% without supplemental oxygen administration at 2 h after birth, were admitted to the Neonatal Intensive Care Unit (NICU). Routine management consisted of supplemental oxygen administration to maintain arterial oxygen saturation levels between 94% and 96% and close monitoring. Infants were diagnosed with RDS and received exogenous surfactant when an inspired oxygen fraction of > 0.5 was required in order to maintain a partial arterial oxygen tension of >50 mmHg, in the presence of radiographic signs suggestive of RDS (diffuse, fine granular opacification in both lung fields); these infants were assigned to the RDS group of our study (cases). The non-RDS group (controls) included healthy late-preterm newborns cared for at the local maternity unit during the same period. Late-preterm infants with other respiratory disorders (transient tachypnea of the newborn [defined as respiratory distress that did not meet the RDS criteria and improved in the course of the first 24–48 hours after birth], pneumonia, pneumothorax, diaphragmatic hernia), suspected or proven sepsis, and those with congenital anomalies, were excluded from the study. A group of healthy term infants (same exclusion criteria) was also included as a reference group, in order to assess the distribution of SP-A gene polymorphisms in the general population. This group was also recruited in the local maternity unit during the same period.

Maternal demographics, pregnancy characteristics, and perinatal data were collected by reviewing the obstetrical and neonatal medical files. Blood samples were obtained via peripheral venipuncture during routine blood sampling in the NICU (RDS group) or during the universal neonatal screening process (controls). All participants were recruited after birth and a written informed consent was obtained from the parents prior to blood sampling. The study protocol was approved by the Ethics Committee of the University Hospital of Patras.

### Genotyping of SP genes

#### Genomic DNA extraction

Whole blood samples (1–2 ml) were stored at -80°C in EDTA vacutainer tubes until DNA extraction. Genomic DNA was extracted by using the QIAamp DNA Blood Midi Kit according to the protocol (Qiamp spin columns) provided by the manufacturer (Blood midi kit Qiagen, QIAGEN Inc., Germantown, MD). Genomic DNA was analyzed by electrophoresis on agarose gel to determine quality and relative quantity. SP-A genetic variants (“S1 Table”) and the SP-B Ile131Thr gene polymorphism (“S2 Table”) (were detected using PCR and direct sequencing.

#### PCR amplification

Two sets of primers were designated and subsequently two PCR products were developed for each of SP-A gene. Specifically, for SP-A1 gene, polymorphisms at amino acid (aa) 19, aa50, aa62 were determined in one PCR product and polymorphisms at aa133 and aa219 in another PCR product. For SP-A2 gene, polymorphisms at aa9, aa91 were determined in one PCR product and polymorphisms at aa140 and aa223 in another PCR product. Previously described primers [14] were used for genotyping the SP-B Ile131Thr gene polymorphism. A detailed description of these primers is presented in “S3 Table”. Polymerase chain reactions amplification had a total reaction volume of 50 μl containing 50ng genomic DNA, 0.25 mM of each primer, 0.25μM dNTPs, 2.5units Taq DNA polymerase (Invitrogen^™^, Thermo Fisher Scientific Inc., Waltham, MA). A detailed description of the PCR conditions is presented in “S4 Table”. PCR products were analysed on agarose gel against to known DNA size markers.

#### Sequencing of PCR products

PCR products were submitted to purification followed by direct sequencing, which was outsourced. Specific primers for SP-A and SP-B genes are shown in “S5 Table”.

### Statistical analysis

Assignment of SP-A haplotypes (“S1 Table”) and Hardy-Weinberg equilibrium (HWE) test for all polymorphisms were performed using PLINK (1.07) [22]. Categorical variables were presented as number of cases with frequencies, and compared using the chi-square or Fisher exact test. Continuous variables were presented as medians with ranges, and compared using the Mann-Whitney U test. Only haplotypes with a frequency of >2% were included in the analysis. Univariate logistic regression was used to explore the effect of various haplotypes (SP-A) and genotypes (SP-B) on the development of RDS, while the combined effect of selected perinatal factors and high-risk haplotypes was assessed by multivariable logistic regression analysis. Post-hoc analysis was performed to estimate the power of the associations for the calculated haplotype frequencies at an alpha error of 0.05. Statistics were performed using IBM SPSS software version 20.0 (IBM Corp., Armonk, NY). A p value of <0.05 was considered significant in all instances.

## Results

A study flow diagram is presented in “S1 Fig”. A total of 296 late-preterm infants were born in our hospital during the study period. Fifty nine newborns with RDS fulfilled the inclusion criteria; the parents of 3 of these neonates did not consent to participate in the study. Of the remaining 237 late-preterm infants, 64 presented various morbid conditions (“S6 Table”) and were *a priori* excluded. In 73 late-preterm newborns blood sampling was not possible due to the inconvenient timing of the universal neonatal screening (i.e., blood sampling for neonatal screening was performed at a time when the responsible investigator was not present), while 40 infants were also excluded because their parents did not consent to participate. Thus, the final study population consisted of 56 late-preterm infants with RDS and 60 without RDS. Sixty one healthy term infants were also included as reference population. The characteristics of the two study groups are presented in Table 1. No differences were found between cases and controls in terms of sex, gestational age, birth weight, mode of delivery, complications of pregnancy, and antenatal administration of corticosteroids (Table 1).

**Table 1:** Characteristics of the study groups.

	RDS(N = 56)	non-RDS (N = 60)	p-value
Male gender	34 (60.7)	34 (56.7)	0.658
Cesarean section	40 (71.4)	38 (63.3)	0.353
Gestational age, weeks	36 (34^0/7^–36^6/7^)	36 (34^0/7^–36^6/7^)	0.912
Gestational age <35 weeks	11 (19.6)	12 (20.0)	0.858
Birth weight, g	2680 (2170–3540)	2490 (1500–3330)	0.069
SGA	1 (1.8)	4 (6.7)	0.366
Apgar score 1^st^ minute	8 (5–9)	9 (6–10)	<0.001
Apgar score 5^th^ minute	9 (7–10)	10 (7–10)	<0.001
Multiple gestation	7 (12.5)	14 (23.3)	0.130
Pregnancy complications	19 (33.9)	14 (23.3)	0.206
HDP*	5 (8.9)	4 (6.7)	0.705
Gestational diabetes	2 (3.6)	3 (5.0)	0.894
Antepartum Hemorrhage†	9 (16.1)	4 (6.7)	0.144
Infection‡	3 (5.4)	3 (5.0)	0.931
Maternal diseases§	6 (10.7)	1 (1.7)	0.055
Greek ethnicity	52 (92.9)	55 (91.7)	0.811
Antenatal corticosteroids	22 (39.3)	17 (28.3)	0.212

Data expressed as number of cases (%) or median (range).

Statistical significance was tested by Mann-Whitney U or chi-square/Fisher exact test, as appropriate.

SGA, small for gestational age; HDP, hypertensive disorders of pregnancy

* pregnancy-induced hypertension or eclampsia;

^†^ placenta praevia or placenta abruption;

^‡^ maternal pyrexia or amniotic infection;

^§^ preexisting lung, cardiac or renal disease.

The SP-A1 and SP-A2 genes are linked, their intragenic haplotypes were shown to be in strong linkage disequilibrium [23] and the observed genotype frequencies did not deviate from the expected Hardy-Weinberg distributions (data not shown). There were no differences in the distribution of SP-A1 and SP-A2 haplotypes between the population of late-preterm infants and the reference population of healthy term newborns ([haplotype: Pearson chi-square, p-value] 6A: 0.0005, 0.983; 6A^2^: 0.521, 0.481; 6A^3^: 0.220, 0.639; 6A^4^: 0.915, 0.339; 1A: 0.027, 0.871; 1A^0^: 0.033, 0.857; 1A^1^: 0.614, 0.433; 1A^2^: 0.055, 0.815; 1A^5^: 0.133, 0.715). Similarly, there were no differences in the distribution of the SP-B Ile131Thr genotypes (Thr/Thr, Ile/Thr, Ile/Ile) between late-preterm infants and the reference population (Pearson chi-square: 0.001, p-value: 0.977).

**Fig 1:**
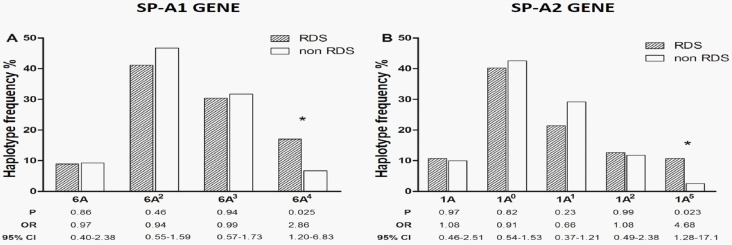
Distribution of SP-A1 and SP-A2 haplotypes in the two late-preterm study groups.

The most prevalent SP-A1 and SP-A2 haplotypes in the two late-preterm groups are presented in Fig 1. The SP-A1 6A^4^ haplotype and the SP-A2 1A^5^ haplotype were significantly overrepresented in newborns with RDS as compared to controls. No differences in the frequency of the other SP-A1 and SP-A2 haplotypes were noted (Fig 1).

Univariate analysis showed that carriers of the SP-A1 6A^4^ haplotype (i.e. homozygous and/or heterozygous status, n = 24, 20.7%) had a 3.3-fold (95% CI 1.25–8.73) higher probability of RDS (P = 0.016, estimated power 74.1%); this probability was 3.4-fold higher for the SP-A1 6A^4^ heterozygous status (95% CI 1.21–9.55) and 2.72-fold higher for the homozygous status (95% CI 0.24–31.1) (multiple comparisons, Table 2). Similarly, the carriers of the SP-A2 1A^5^ haplotype (only heterozygous status in our cohort, n = 15, 12.9%) had a 5.18-fold (95% CI 1.38–19.5) higher probability for RDS (P = 0.015, estimated power 80.0%, Table 2). Overall, the SP-A1 6A^4^ or/and SP-A2 1A^5^ haplotype was present in 20 newborns with RDS (35.7%) and in 7 without RDS (11.7%), resulting in a 4.21-fold (95% CI 1.61–11.0) higher probability of RDS in carriers of these haplotypes (P = 0.003, estimated power 89.9%). The distribution of the SP-B Ile131Thr genotypes was similar between the two late-preterm groups (Table 3). No allele of the SP-B Ile131Thr polymorphism was found to influence the effect of SP-A haplotypes on the development of RDS (“S7 Table”).

**Table 2:** Associations between SP-A1 and SP-A2 haplotypes and RDS.

SP-A1 haplotype	RDSn (%)	Controls n (%)	OR	95% CI	SP-A2 haplotype	RDS n (%)	Controls n (%)	OR	95% CI
**6A**					**1A**				
X/X	47 (83.9)	51 (85.0)	Ref.		X/X	45 (80.3)	50 (83.4)	Ref.	
6A/X	8 (14.3)	7 (11.7)	1.24	0.42–3.69	1A/X	10 (17.9)	8 (13.3)	1.39	0.50–3.83
6A/6A	1 (1.8)	2 (3.3)	0.54	0.05–6.18	1A/1A	1 (1.8)	2 (3.4)	0.56	0.49–6.34
**6A**^**2**^					**1A**^**0**^				
X/X	21 (37.5)	15 (25.0)	Ref.		X/X	22 (39.3)	18 (30.0)	Ref.	
6A^2^/X	24 (42.9)	34 (56.7)	0.50	0.22–1.17	1A^0^/X	23 (41.1)	33 (55.0)	0.57	0.25–1.29
6A^2^/6A^2^	11 (19.6)	11 (18.3)	0.71	0.25–2.08	1A^0^/1A^0^	11 (19.6)	9 (15.0)	1.00	0.34–2.94
**6A**^**3**^					**1A**^**1**^				
X/X	28 (50.0)	29 (48.3)	Ref.		X/X	35 (62.5)	28 (46.7)	Ref.	
6A^3^/X	22 (39.3)	24 (40.0)	0.95	0.44–2.07	1A^1^/X	18 (32.1)	29 (48.3)	0.50	0.23–1.07
6A^3^/6A^3^	6 (10.7)	7 (11.7)	0.89	0.27–2.97	1A^1^/1A^1^	3 (5.4)	3 (5.0)	0.80	0.15–4.27
**6A**^**4**^					**1A**^**2**^				
X/X	39 (69.6)	53 (88.3)	Ref.		X/X	43 (76.8)	49 (81.7)	Ref.	
6A^4^/X	**15 (26.8)**	**6 (10.0)**	**3.40**	**1.21–9.55**	1A^2^/X	12 (21.4)	8 (13.3)	1.71	0.64–4.57
6A^4^/6A^4^	2 (3.6)	1 (1.7)	2.72	0.24–31.1	1A^2^/1A^2^	1 (1.8)	3 (5.0)	0.38	0.04–3.79
					**1A**^**5**^				
					X/X	44 (78.6)	57 (95.0)	Ref.	
					1A^5^/X	**12 (21.4)**	**3 (5.0)**	**5.18**	**1.38–19.5**
					1A^5^/1A^5^	-	-	-	-

Logistic regression analyses using the genotypes (no, heterozygote, homozygote) of the most common SP-A1 and SP-A2 haplotype as independent variables.

X = any haplotype other than the haplotype of interest.

**Table 3:** Associations between SP-B Ile131Thr genotypes and RDS.

Genotype	RDS n (%)	Controls n (%)	OR	95% CI
Ile/Ile	15 (26.8)	13 (21.7)	Ref.	
Ile/Thr	28 (50.0)	37 (61.6)	0.66	0.27–1.60
Thr/Thr	13 (23.2)	10 (16.7)	1.13	0.37–3.42

Multivariable regression analysis revealed that the negative effect of the SP-A1 6A^4^ and SP-A2 1A^5^ haplotypes was preserved when adjusting for known risk or protective factors, such as male gender, smaller gestational age, smaller size (weight) for gestational age, complications of pregnancy and administration of antenatal corticosteroids, with the carriers of the 6A^4^ and 1A^5^ haplotypes having a 4- and 5-fold higher probability for RDS, respectively (Table 4). The presence of both SP-A1 6A^4^ and SP-A2 1A^5^ haplotypes (n = 13) was not associated with a higher risk for RDS as compared to the presence of either haplotype alone (n = 14) (Table 4).

**Table 4:** Combined effect of selected perinatal factors and SP-A1 6A^4^ and SP-A2 1A^5^ haplotypes on the probability for RDS.

	Model 1	Model 2	Model 3
Male gender	1.04 (0.47–2.30)	1.14 (0.51–2.51)	0.96 (0.42–2.16)
Gestational age	0.79 (0.50–1.23)	0.86 (0.56–1.34)	0.81 (0.52–1.28)
SGA	0.14 (0.01–1.68)	0.18 (0.02–2.03)	0.12 (0.01–1.53)
Pregnancy complications*	1.47 (0.61–3.54)	1.41 (0.58–3.39)	1.45 (0.59–3.56)
Antenatal steroids	2.21 (0.87–5.64)	1.76 (0.70–4.43)	2.32 (0.90–6.00)
Ethnicity	0.92 (0.20–4.20)	0.83 (0.19–3.60)	0.85 (0.19–3.94)
SP-B Ile131Thr polymorphism†	0.94 (0.54–1.64)	0.96 (0.55–1.67)	0.94 (0.54–1.66)
SP-A haplotype:			
SP-A1 6A^4^	**4.01 (1.39–11.6)**	-	-
SP-A2 1A^5^	-	**5.10 (1.26–20.8)**	-
SP-A1 6A^4^ or SP-A2 1A^5^	-		**5.44 (1.44–20.5)**
SP-A1 6A^4^ and SP-A2 1A^5^	-		**4.83 (1.11–21.7)**

Data are adjusted ORs (95% CI) derived from multivariable logistic regression analysis (three separate models according to the SP-A haplotype)

* hypertensive disorders of pregnancy, gestational diabetes, antepartum hemorrhage, infection,

^†^ threonine allele

SGA, small for gestational age.

## Discussion

In this study we investigated whether specific SP-A and SP-B gene polymorphisms are associated with RDS in late-preterm newborns. The main finding was that the SP-A1 6A^4^ and SP-A2 1A^5^ haplotypes were significantly overrepresented in infants who developed RDS as compared to late-preterm controls. Conversely, there were no differences in the frequency of other SP-A1 and SP-A2 haplotypes or the SP-B Ile131Thr polymorphism alleles. Late-preterm carriers of the 6A^4^ and/or 1A^5^ haplotype(s) had a significantly higher probability of RDS, and—more important—this effect was independent of known risk factors such as male sex, smaller gestational age, and pregnancy complications, or the protective effect of antenatal corticosteroids.

Previous reports point towards a direct but extremely variable link between specific SP-A genetic variants and development of RDS in preterm neonates. In a Finnish population of infants born at <32 weeks of gestation, the SP-A1 6A^2^ and SP-A2 1A^0^ haplotypes have been shown to predispose to RDS, while the SP-A1 6A^3^, SP-A2 1A^1^ and SP-A2 1A^2^ haplotypes were protective in homozygous status [13]. In a subsequent study from the same group, the negative effect of the SP-A1 6A^2^ haplotype was confirmed for very preterm singleton infants, although 6A^2^ was protective for multiples born at >32 weeks of gestation [18]. A family-based linkage study from North America demonstrated that the SP-A1 6A^2^ and SP-A2 1A^0^ haplotypes may increase the susceptibility to RDS in preterm neonates [15]; on the contrary, the SP-A2 1A^0^ haplotype had a protective effect against RDS in a study of Korean preterm newborns [16]. Differences in the studied populations in terms of gestational age, analysed number of siblings (i.e., twins or singleton), race, and ethnicity, might explain the above inconsistencies.

The results of our study are not in line with the findings of the above reports; the SP-A1 6A^4^ and SP-A2 1A^5^ haplotypes were strongly related to RDS in our population (Fig 1, Tables 2 and 4, “S7 Table”), while the SP-A1 6A^2^ and SP-A2 1A^1^ haplotypes showed only a tendency towards protection (Fig 1, Table 2, “S7 Table”). It should be noted, however, that our study cohort consisted solely of late-preterm infants, a population that has not been studied previously [13, 15, 16, 18]; it has been suggested that the complex cross-talk between structural lung maturity and SP-A genetic variants may present gestational-age variations that influence the development of RDS in preterm neonates [14]. In addition, the vast majority of the participants in our study were of Greek ethnicity (Table 1). Given the higher prevalence of RDS in Greek late-preterm infants [24], it is tempting to speculate that the aforementioned discrepancy between our findings and those of previous reports [13, 15, 16, 18] could be attributed to the different genetic profile of our population. Similar inconsistencies regarding the role of specific SP-A haplotypes between different populations, have been reported previously [16].

The SP-B Ile131Thr polymorphism (i.e, the threonine allele) has been also associated with high risk of RDS in the first-born preterm twin [17], or has been shown to influence the association between certain SP-A1/SP-A2 haplotypes and RDS suggesting the existence of a gene-gene interaction mechanism [14, 18, 19]. In our study, we found no association between the SP-B Ile131Thr polymorphism and RDS (Tables 3 and 4); similarly, our results were not suggestive of an interaction between SP-A and SP-B genes with regards to the development of RDS (Table 4, “S7 Table”). The particular characteristics of our cohort may explain the discrepancies between our findings and those of previous studies.

The exact molecular mechanisms underlying the association between SP-A genetic variants and RDS remain only partially understood. It is well known that the SP-A gene locus exhibits variability which may affect the quantity and quality of the gene product [25]. For example, it has been shown that the substitution of the hydrophilic amino acid arginine by the hydrophobic tryptophan within the SP-A1 gene coding sequence (i.e., the SP-A1 6A^4^ haplotype) may change the biochemical properties of the protein and alter its *in vitro* behavior, predisposing to the development of idiopathic pulmonary fibrosis [20]. Although similar data regarding RDS are lacking, it is tempting to speculate that specific changes within the SP-A coding region(s) may affect the structure and as a consequence the function of the protein and predispose to the development of the disease. Interestingly, the SNPs that determine the SP-A1 6A^4^ and SP-A2 1A^5^ haplotypes (i.e., those which were found to be associated with RDS in the present study) result in amino acid substitutions within the collagen-like and the carbohydrate recognition domain of the SP-A protein [25], two regions of critical importance for optimal protein function [25].

In our study, the fraction of newborns with RDS who received antenatal corticosteroids was higher than that of controls, a finding which could be attributed to the higher percentage of high-risk gestations (in terms of pregnancy complications and preexisting maternal diseases) in the RDS group (Table 1). Although previous reports have shown that different SP-A haplotypes may be associated with a different response to dexamethasone therapy in vitro [26], the small sample size (i.e., the 39 newborns who had received corticosteroids), as well as the lack of homogeneity in terms of the exact indications, timing and dosing of antenatal corticosteroid administration, do not permit to explore this hypothesis in our study.

The identified high-risk genetic variants of SP-A were not among the most prevalent in our study population (Fig 1, Table 2). The frequency of the SP-A1 6A^4^ and/or SP-A2 1A^5^ haplotype was relatively low (at least one of these haplotypes was present in 27 infants or 23.3% of the study population), but comparable or even higher to that of known risk factors for RDS, such as complications of pregnancy (33 infants or 28.4%), gestational age ≤35 weeks (23 infants or 19.8%), and maternal preexistent morbidity (7 infants or 6%). Therefore, the importance of these SP-A genetic variants should not be underestimated, especially given their clear association with RDS in contrast to the other risk factors mentioned above (Table 4).

The distribution of the SP-A1 and SP-A2 haplotypes in our study population of late-preterm infants was not divergent from that described previously for individuals of Greek ethnicity [23]. Also, the most frequent genetic variants (i.e., the haplotypes SPA-1 6A^2^ and SPA-2 1A^0^) were in accordance with those reported in other neonatal populations in similar studies [10, 13, 15, 18, 27]. However, the different ethnical characteristics and the rather small sample size represents an important limitation of the present work. Although the reported associations between specific SP-A haplotypes and RDS were strong and unaffected by other confounding factors, our findings should be validated in larger late-preterm populations, ideally with variable ethnic background; therefore, our findings cannot be generalized. Larger sample sizes are also required to explore possible associations between SP-A haplotypes and SP-B polymorphisms in relation to the development of RDS in late-preterm neonates, or to assess the influence of specific SP-A genetic variants on the response to antenatal corticosteroids.

In conclusion, the findings of our study suggest that specific SP-A genetic variants (i.e., the SP-A1 6A^4^ and SP-A2 1A^5^ haplotypes) influence the susceptibility to RDS in Greek late-preterm infants, independently of the effect of other perinatal risk factors. Further research is required to clarify the exact mechanism by which these genetic variants predispose to RDS in late-preterm neonates and to develop dedicated preventive strategies for optimizing the respiratory outcomes of this vulnerable population.

## Supporting Information

S1 Fig Flow diagram of the study. (DOCX)

S1 Table SP-A genetic variants or intragenic haplotypes. (DOCX)

S2 Table SP-B Ile131Thr gene polymorphism. (DOCX)

S3 Table Primers used for genotyping SP-B, SP-A1 and SP-A2 genes (PCR). (DOCX)

S4 Table PCR conditions. (DOCX)

S5 Table Primers used for genotyping SP-B, SP-A1 and SP-A2 genes (direct sequencing). (DOCX)

S6 Table Characteristics of the late-preterm neonates excluded from the study due to various morbid conditions. (DOCX)

S7 Table Effect of SP-A haplotypes in the presence of SP-B Ile131Thr polymorphism. (DOCX)
