# Supplementary material for: Surfactant Protein A and B Gene Polymorphisms and Risk of Respiratory Distress Syndrome in Late-Preterm Neonates
Source: PLoS One. 2016 Nov 11;11(11):e0166516. doi: 10.1371/journal.pone.0166516 (PMC5106092; doi:10.1371/journal.pone.0166516)
Supplement: S1 Fig — (DOCX) [file pone.0166516.s001.docx]

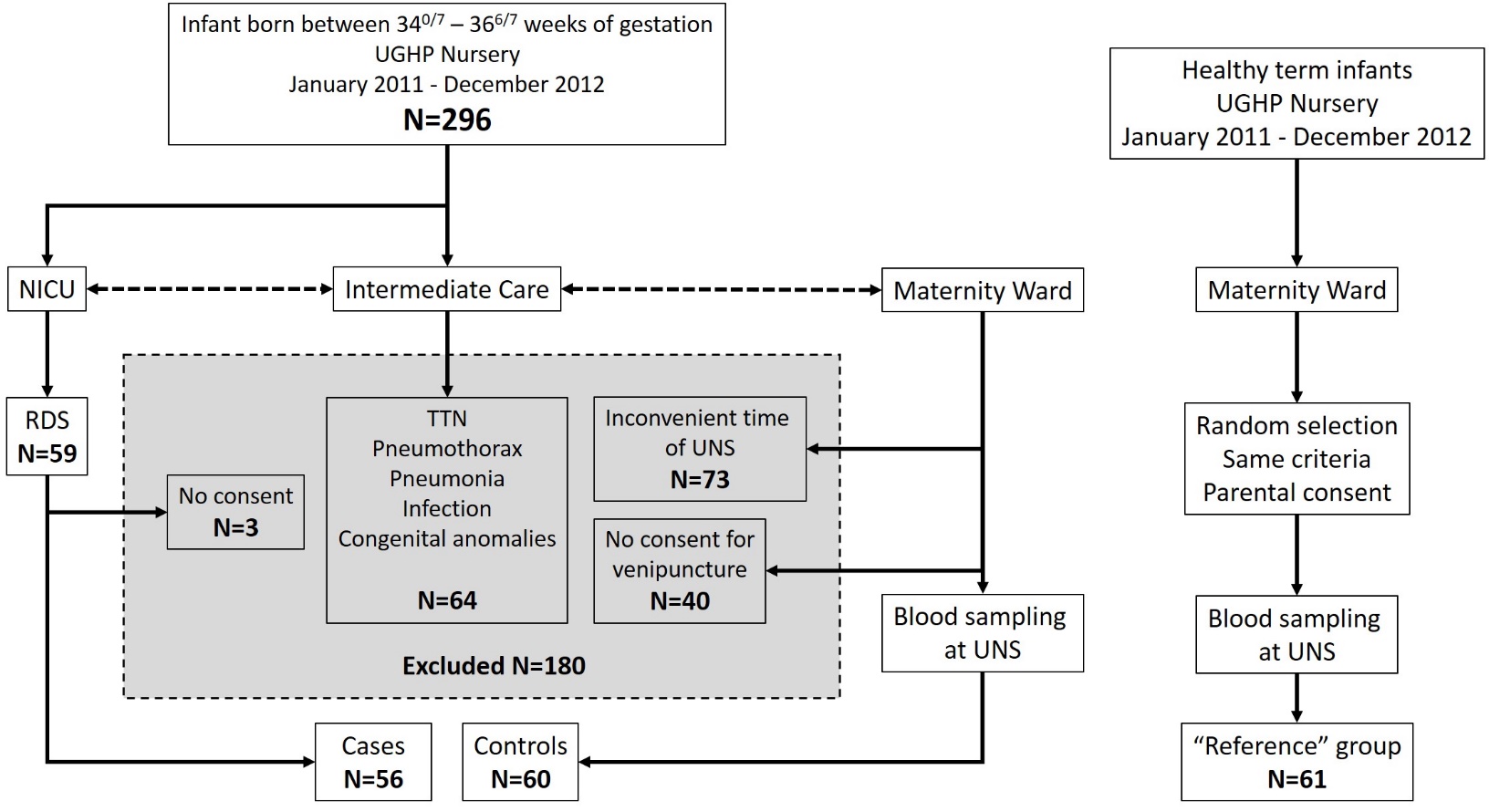


**Figure S1.** Flow diagram of the study

UGHP: University General Hospital of Patras, NICU: Neonatal Intensive Care Unit, RDS: Respiratory Distress Syndrome, TTN: Transient Tachypnea of the Newborn, UNS: Universal Neonatal Screening
