# Supplementary material for: Surfactant Protein A and B Gene Polymorphisms and Risk of Respiratory Distress Syndrome in Late-Preterm Neonates
Source: PLoS One. 2016 Nov 11;11(11):e0166516. doi: 10.1371/journal.pone.0166516 (PMC5106092; doi:10.1371/journal.pone.0166516)
Supplement: S1 Table — (DOCX) [file pone.0166516.s002.docx]

| **Table S1. SP-A genetic variants or intragenic haplotypes** | | | | | | |
| --- | --- | --- | --- | --- | --- | --- |
| **SP-A1 (SFTPA1) GENE** | | | | | | |
| **SNP id^†^** | | rs1059047 | rs1136450 | rs1136451 | rs1059057 | rs4253527 |
| **Allele - Nucleotide (ancestral/mutant)** | | T/C | C/G | A/G | A/G | C/T |
| **Amino acid substitution^*^** | | aa19: Val>Ala | aa50: Leu>Val | aa62: Pro>Pro | aa133: Trp>Trp | aa219:Arg>Trp |
| **SP-A1 haplotype^‡^** | **6A** | C/Ala | C/Leu | G/Pro | G/Trp | C/Arg |
|  | **6Α^2^** | T/Val | G/Val | A/Pro | A/Trp | C/Arg |
|  | **6Α^3^** | T/Val | C/Leu | A/Pro | A/Trp | C/Arg |
|  | **6Α^4^** | T/Val | C/Leu | G/Pro | A/Trp | T/Trp |
| **SP-A2 (SFTPA2) GENE** | | | | | | |
| **SNP id^†^** | | rs1059046 | rs17886395 | rs1965707 | rs1965708 |  |
| **Allele - Nucleotide (ancestral/mutant)** | | C/A | G/C | C/T | C/A |  |
| **Amino acid substitution^*^** | | aa9: Thr>Asn | aa91: Ala>Pro | aa140: Ser>Ser | aa223: Gln>Lys |  |
| **SP-A2 haplotype^‡^** | **1A** | C/ Thr | C/Pro | C/Ser | C/Gln |  |
|  | **1Α^0^** | A/ Asn | G/Ala | C/Ser | C/Gln |  |
|  | **1A^1^** | C/ Thr | G/Ala | T/Ser | A/Lys |  |
|  | **1Α^2^** | C/ Thr | G/Ala | C/Ser | C/Gln |  |
|  | **1A^3^** | A/ Asn | G/Ala | T/Ser | A/Lys |  |
|  | **1A^5^** | C/ Thr | C/Pro | T/Ser | C/Gln |  |
| ^†^SNP id: Single nucleotide polymorphism identity according to NCBI Reference Sequence Database  **^‡^** SP-A1 haplotypes are denoted as 6A^n^ and SP-A2 haplotypes are denoted as 1A^n^  *Assignment of a number to amino acid (aa) position is based on the precursor molecule of SP-A protein | | | | | | |
