# Supplementary material for: Surfactant Protein A and B Gene Polymorphisms and Risk of Respiratory Distress Syndrome in Late-Preterm Neonates
Source: PLoS One. 2016 Nov 11;11(11):e0166516. doi: 10.1371/journal.pone.0166516 (PMC5106092; doi:10.1371/journal.pone.0166516)
Supplement: S2 Table — (DOCX) [file pone.0166516.s003.docx]

| **Table S2. SP-B Ile131Thr gene polymorphism** | | | | |
| --- | --- | --- | --- | --- |
| **SP-B (SFTPB) GENE** | | | | |
| **SNP id^†^** | **Allele - Nucleotide (ancestral/mutant)** | **Amino acid substitution^*^** | **SP-B genotype** | **SP-B allele** |
| rs1130866 | T/C | aa131: Ile >Thr | Ile/Ile (TT) | Isoleucine (T) |
|  |  |  | Ile/Thr (CT) | Threonine (C) |
|  |  |  | Thr/Thr (CC) |  |
| ^†^SNP id: Single nucleotide polymorphism identity according to NCBI Reference Sequence Database  **^*^**amino acid (aa) position | | | | |
