# Supplementary material for: Surfactant Protein A and B Gene Polymorphisms and Risk of Respiratory Distress Syndrome in Late-Preterm Neonates
Source: PLoS One. 2016 Nov 11;11(11):e0166516. doi: 10.1371/journal.pone.0166516 (PMC5106092; doi:10.1371/journal.pone.0166516)
Supplement: S3 Table — (DOCX) [file pone.0166516.s004.docx]

| **Table S3. Primers used for genotyping SP-B, SP-A1 and SP-A2 genes (PCR)** | | | |
| --- | --- | --- | --- |
| **Gene** | **SNP** | **Primer ID** | **Sequence** |
| **SP-B** | Ile131Thr | Forward: SPBTaaF1  Reverse: SPBΔi4R | 5'-TGGGGGATTAGGGGTCAGTC-3'  5'-GCTGAAGGGCACGTAGTTTC-3' |
| **SP-A1** | Val19Ala  Val50Leu  Pro62 | Forward: SPA1-19/50/62F  Reverse: SPA1-19/50/62R | 5'-AGTGAGTGAGTGACCTGACTAA-3'  5'-CCACACTCTCCAGGGATACCA-3' |
|  | Thr 133  Arg219Trp | Forward: SPA1-133/219F  Reverse: SPA1-133/219R | 5'-GGCCCAGAGGAGACAGAAGC-3'  5'-CCTGTCCCATGGCCTAAAT-3' |
| **SP-A2** | Asn9Thr  Ala91Pro | Forward: SPA2-9/91F  Reverse: SPA2-9/91R | 5'-TGAGTGACCTCATCCTTCCTT-3'  5'-CTGACTTCAGGTCGCTGTGC-3' |
|  | Ser140  Gln223Lys | Forward: SPA2-140/223F  Reverse: SPA2-140/223R | 5'-GGCCAAGAGGAGACAAGCAG-3'  5'-CCTGTCCCATGGCCTAAAT-3' |
