# Supplementary material for: Surfactant Protein A and B Gene Polymorphisms and Risk of Respiratory Distress Syndrome in Late-Preterm Neonates
Source: PLoS One. 2016 Nov 11;11(11):e0166516. doi: 10.1371/journal.pone.0166516 (PMC5106092; doi:10.1371/journal.pone.0166516)
Supplement: S4 Table — (DOCX) [file pone.0166516.s005.docx]

| **Table S4. PCR conditions** | | | | | | |
| --- | --- | --- | --- | --- | --- | --- |
| **Gene** | **SNP** | **Primer** | **Step** | | **Temp**$\boldsymbol{^{\circ}}$**C** | **Time** |
| **SP-B** | Ile131Thr | F:SPBTaaF1  R :SPBΔi4R | 1. Initial Denaturation | | 94 | 7 min |
|  |  |  | PCR cycle | 2. Denature | 94 | 30 sec |
|  |  |  |  | 3. Anneal | 64 | 35 sec |
|  |  |  |  | 4. Extend | 72 | 90 sec |
|  |  |  | 5. Repeat step 2, 3, 4 for 29 cycles | | - | - |
|  |  |  | 6. Final Extension | | 72 | 7 min |
| **SP-A1** | Val19Ala  Val50Leu  Pro62 | F: SPA1-19/50/62F R: SPA1-19/50/62R | 1. Initial Denaturation | | 94 | 7 min |
|  |  |  | PCR cycle | 2. Denature | 94 | 30 sec |
|  |  |  |  | 3. Anneal | 57 | 40 sec |
|  |  |  |  | 4. Extend | 72 | 90 sec |
|  |  |  | 5. Repeat step 2, 3, 4 for 29 cycles | | - | - |
|  |  |  | 6. Final Extension | | 72 | 7 min |
|  | Thr133  Arg219Trp | F: SPA1-133/219F R: SPA1-133/219R | 1. Initial Denaturation | | 94 | 7 min |
|  |  |  | PCR cycle | 2. Denature | 94 | 30 sec |
|  |  |  |  | 3. Anneal | 60 | 40 sec |
|  |  |  |  | 4. Extend | 72 | 90 sec |
|  |  |  | 5. Repeat step 2, 3, 4 for 29 cycles | | - | - |
|  |  |  | 6. Final Extension | | 72 | 7 min |
| **SP-A2** | Asn9Thr  Ala91Pro | F: SPA2-9/91F R: SPA2-9/91R | 1. Initial Denaturation | | 94 | 7 min |
|  |  |  | PCR cycle | 2. Denature | 94 | 30 sec |
|  |  |  |  | 3. Anneal | 59 | 40 sec |
|  |  |  |  | 4. Extend | 72 | 120 sec |
|  |  |  | 5. Repeat step 2, 3, 4 for 29 cycles | | - | - |
|  |  |  | 6. Final Extension | | 72 | 7 min |
|  | Ser140  Gln223Lys | F:SPA2-140/223F R:SPA2-140/223R | 1. Initial Denaturation | | 94 | 7 min |
|  |  |  | PCR cycle | 2. Denature | 94 | 30 sec |
|  |  |  |  | 3. Anneal | 60 | 40 sec |
|  |  |  |  | 4. Extend | 72 | 90 sec |
|  |  |  | 5. Repeat step 2, 3, 4 for 31 cycles | | - | - |
|  |  |  | 6. Final Extension | | 94 | 7 min |
