# Supplementary material for: Surfactant Protein A and B Gene Polymorphisms and Risk of Respiratory Distress Syndrome in Late-Preterm Neonates
Source: PLoS One. 2016 Nov 11;11(11):e0166516. doi: 10.1371/journal.pone.0166516 (PMC5106092; doi:10.1371/journal.pone.0166516)
Supplement: S6 Table — (DOCX) [file pone.0166516.s007.docx]

| **Table S6.** Characteristics of the late-preterm neonates excluded from the study (N=64) due to various morbid conditions | |
| --- | --- |
|  |  |
| Gestational age, weeks [median (range)] | 36 (34^0/7^-36^6/7^) |
| Morbidities* |  |
| TTN | 47 |
| Pneumonia | 3 |
| Pneumothorax | 3 |
| Diaphragmatic hernia | 1 |
| Sepsis | 16 |
| Congenital anomalies | 5 |
| Antenatal corticosteroids | 21 (32.8) |
| Data expressed as number of cases (%).  * 11 neonates presented more than one conditions | |
