# Supplementary material for: Surfactant Protein A and B Gene Polymorphisms and Risk of Respiratory Distress Syndrome in Late-Preterm Neonates
Source: PLoS One. 2016 Nov 11;11(11):e0166516. doi: 10.1371/journal.pone.0166516 (PMC5106092; doi:10.1371/journal.pone.0166516)
Supplement: S7 Table — (DOCX) [file pone.0166516.s008.docx]

| **Table S7. Effect of SP-A haplotypes in the presence of SP-B Ile131Thr polymorphism** | | | | | |
| --- | --- | --- | --- | --- | --- |
| **Haplotypes** | **Unadjusted** | |  | **Adjusted for SP-B genotypes (Ile/Ile, Ile/Thr, or Thr/Thr)** | |
|  | **OR** | **95% CI** |  | **OR** | **95% CI** |
| **SP-A1** |  |  |  |  |  |
| **6A** | 1.09 | 0.40-2.97 |  | 1.08 | 0.40-2.96 |
| **6Α^2^** | 0.56 | 0.25-1.23 |  | 0.56 | 0.25-1.95 |
| **6Α^3^** | 0.94 | 0.45-1.94 |  | 0.94 | 0.45-1.95 |
| **6Α^4^** | 3.30 | 1.25-8.73 |  | 3.30 | 1.25-8.73 |
| **SP-A2** |  |  |  |  |  |
| **1A** | 1.22 | 0.48-3.15 |  | 1.22 | 0.47-3.15 |
| **1Α^0^** | 0.66 | 0.31-1.43 |  | 0.66 | 0.30-1.42 |
| **1Α^1^** | 0.53 | 0.25-1.10 |  | 0.52 | 0.25-1.10 |
| **1A^2^** | 1.35 | 0.55-3.32 |  | 1.35 | 0.55-3.33 |
| **1A^5^** | 5.18 | 1.38-19.5 |  | 5.19 | 1.38-19.6 |
| Logistic regression models | | | | | |
